# High thrombin-activatable fibrinolysis inhibitor expression in thrombi from stroke patients in elevated estrogen states

**DOI:** 10.1186/s12883-024-03579-2

**Published:** 2024-03-07

**Authors:** Tamanna Agarwal, Oana Madalina Mereuta, Sherief Ghozy, Jorge L Arturo Larco, Cem Bilgin, Ram Kadirvel, Waleed Brinjikji, David F Kallmes

**Affiliations:** 1 Faculty of Medicine in Hradec Kralove, Charles University, Prague, Czech Republic; 2 Department of Radiology, Mayo Clinic, 200 First St SW, Rochester, MN 55905 USA; 3 Department of Neurologic Surgery, Mayo Clinic, Rochester, MN USA; 4 Henry Ford Hospital, Detroit, MI USA

**Keywords:** Stroke, Thrombectomy, TAFI, Estrogen, OCP.

## Abstract

**Background:**

The risk of acute ischemic stroke (AIS) associated with high estrogen states, including pregnant patients and those using oral contraceptives, has been well documented. We described the histological composition of thrombi collected in these cases.

**Methods:**

From a prospective tissue registry (STRIP registry) of thrombi retrieved during mechanical thrombectomy for AIS, we identified 5 patients with high estrogen states: 1 post-partum patient, 1 undergoing hormone replacement therapy and 3 consuming oral contraceptive pills. Five male control patients were randomly chosen matched by age. Immunohistochemistry for CD42b (platelets), von Willebrand factor (vWF), thrombin-activatable fibrinolysis inhibitor (TAFI), fibrinogen and plasminogen activator inhibitor-1 (PAI-1) was performed. Expression was quantified using Orbit Image Software. Student’s t-test was performed as appropriate.

**Results:**

Mean TAFI content for the high estrogen state group was higher than controls (25.6 ± 11.9% versus 9.3 ± 9.0%, *p* = 0.043*). Mean platelet content for the high estrogen state group was lower than controls (41.7 ± 10.6% versus 61.8 ± 12.9%, *p* = 0.029*). No significant difference was found in vWF, fibrinogen and PAI-1 expression. Mean time to recanalize was higher in the high estrogen state group compared to the control group (57.8 ± 27.6 versus 22.6 ± 11.4 min, *p* = 0.0351*). The mean number of passes required was higher in the high estrogen group compared to controls 4.6 versus 1.2, *p* = 0.0261*).

**Conclusions:**

TAFI expression, a powerful driver of thrombosis, was significantly higher in stroke thrombi among patients with high estrogen states compared to controls.

## Introduction

High estrogen states, arising from exogenous estrogen use i.e. oral contraception or hormone replacement therapy, and related to pregnancy and the post-partum period, have been well established as settings wherein the absolute risk of acute ischemic stroke (AIS) is higher in comparison to the general population [1, 2]. While it has been well established that estrogen increases levels of procoagulant factors, its impact on the fibrinolytic system has recently become of interest [1, 3, 4]. Fibrinolytic activity should be increased after estrogenic treatment via decreased levels of plasminogen activator inhibitor (PAI-1) leading to increased levels of tissue plasminogen activator (tPA). However, thrombin activatable fibrinolysis inhibitor (TAFI) counterbalances fibrinolysis generating a net prothrombotic state [5]. Notably, increased levels of TAFI have been associated with an increased risk of acute ischemic stroke (AIS) [6–8].

Currently, to our knowledge, there are no studies describing the composition of cerebral thrombi retrieved from large vessel occlusion (LVO) AIS patients undertaking estrogenic treatment in the form of oral contraceptive pills (OCPs) or hormone replacement therapy (HRT), or in the hyperestrogenic prothrombotic setting of pregnancy. In this study, we aim to characterize the histological and immunohistochemical features of large vessel occlusions developing in patients in previously defined hyperestrogenic states.

## Methods

This study was performed as a part of the multi-institutional Stroke Thromboembolism Registry of Imaging and Pathology (STRIP). The waiver of consent was granted and this study was approved by the Institutional Review Board (IRB 16-001131) at Mayo Clinic (Rochester, Minnesota). All methods were carried out in accordance with relevant institutional guidelines and regulations. Clinical data were reviewed for 150 female patients enrolled in the STRIP registry from Mayo Clinic between 2017 and 2022. Patients were included in this study if they were adult women, exposed to exogenous estrogen in the form of HRT or OCPs, pregnant or early post-partum at the time of stroke, underwent mechanical thrombectomy to treat AIS, and had embolic material available for analysis.

A total of 5 women were included in the high estrogen state group: three users of OCPs, one user of HRT and one patient with one week post-partum AIS. Further, a control group of 5 male patients enrolled in STRIP was matched based on age.

### Clot processing and histology

**Fig. 1:**
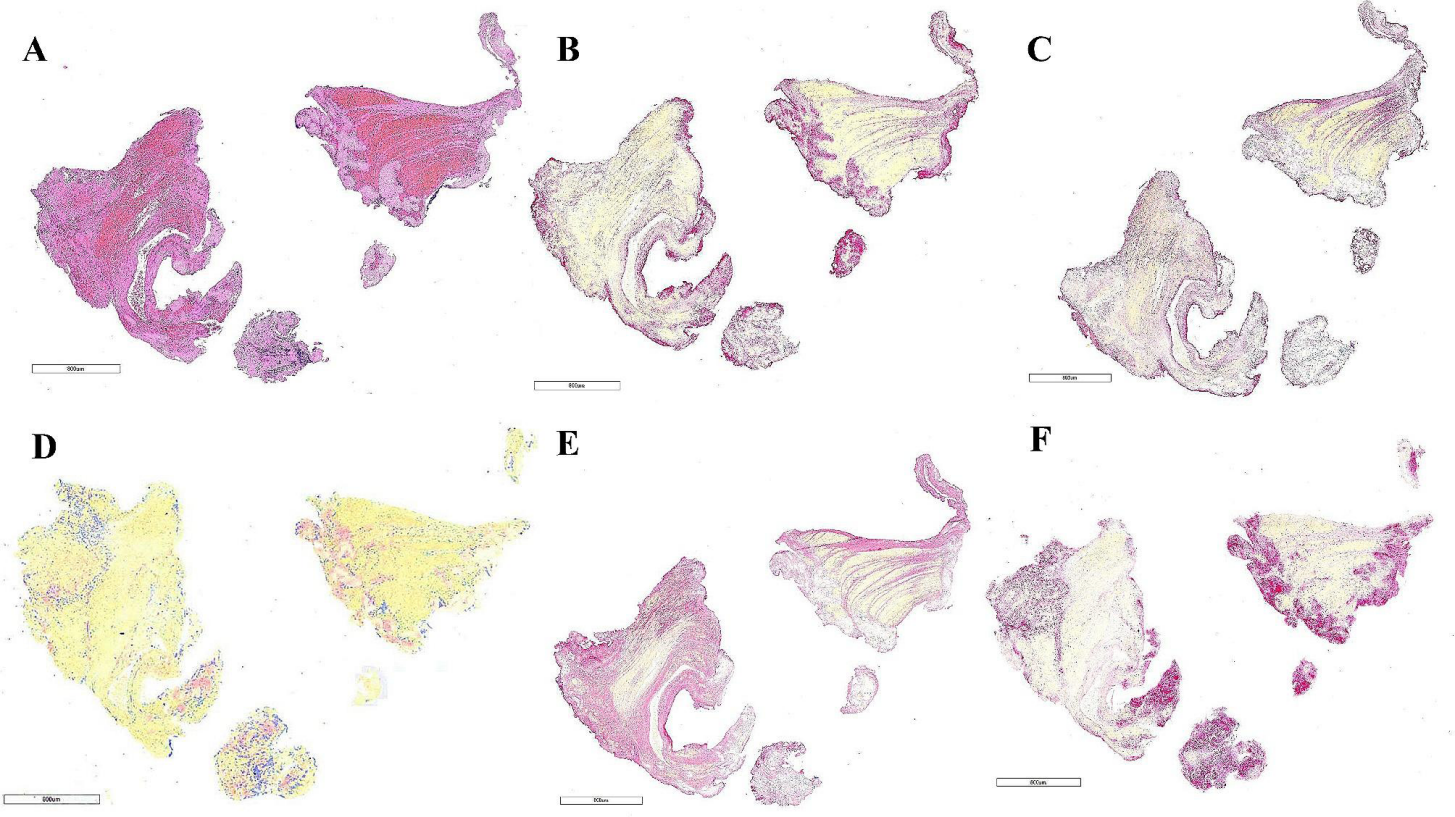
Representative slides from high estrogen state group Thrombus composition in a representative case of acute ischemic stroke associated with OCP use (patient 3). H&E staining showing fibrin/platelet aggregates (pink) and red blood cells (red) seen on(**A**). Immunostaining for platelets (CD42b) (**B**, red), von Willebrand Factor (**C**, pink), thrombin-activatable fibrinolysis inhibitor, TAFI (**D**, pink), fibrinogen (**E**, pink) and plasminogen activator inhibitor, PAI-1 (**F**, pink) Scale bar (**A-F**) = 800 mm.

**Fig. 2:**
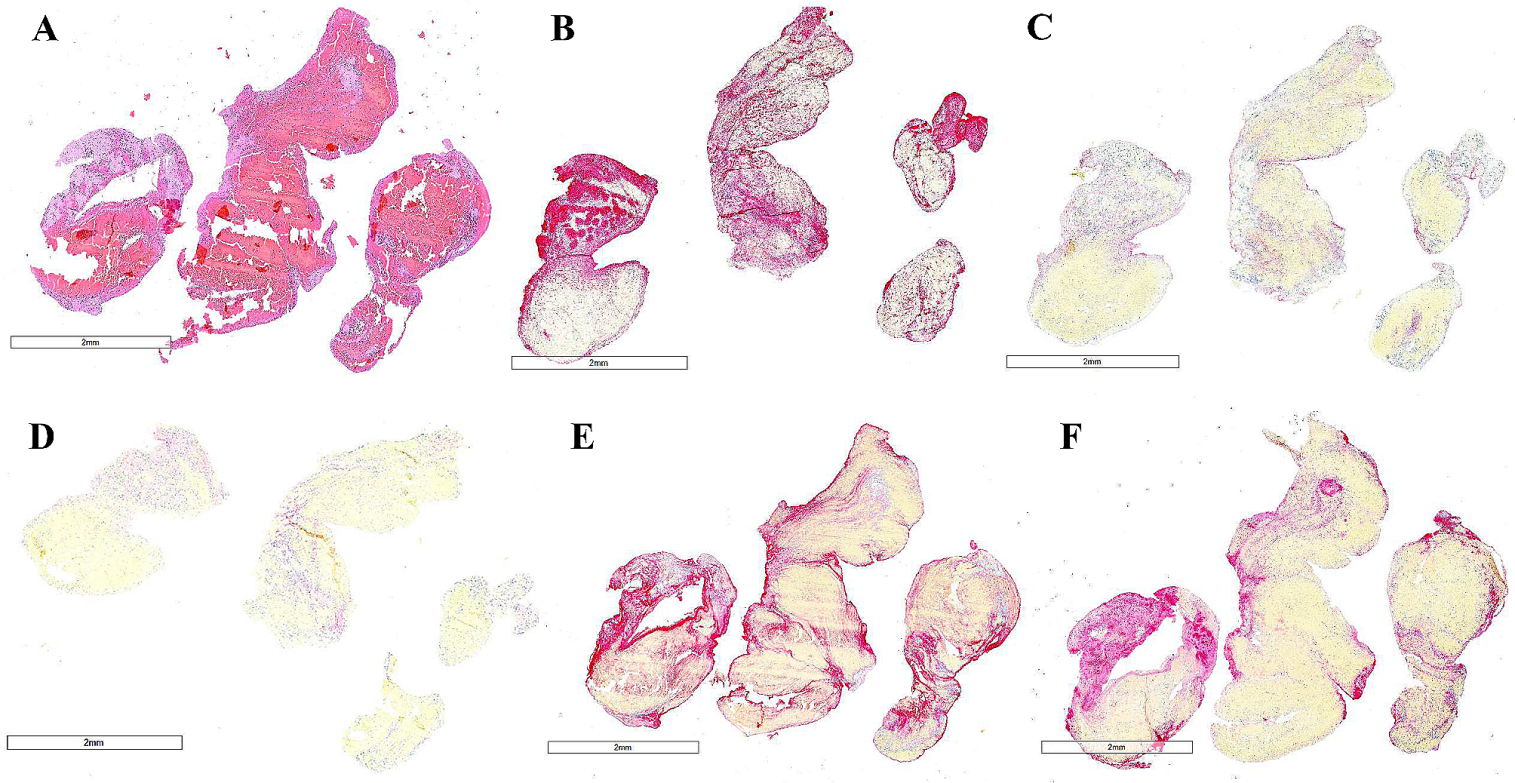
Representative slides from control group Thrombus composition in a representative control case of acute ischemic stroke (control patient 3). H&E staining showing fibrin/platelet aggregates (pink) and red blood cells (red) seen on(**A**). Immunostaining for platelets (CD42b) (**B**, red), von Willebrand Factor (**C**, pink), thrombin-activatable fibrinolysis inhibitor, TAFI (**D**, pink), fibrinogen (**E**, pink) and plasminogen activator inhibitor, PAI-1 (**F**, pink) Scale bar (**A-F**) = 2 mm.

Once received, each embolus was immediately fixed in 10% neutral buffer formalin, followed by standard tissue processing protocol and embedding in paraffin. The embedded clots were cut into 3 μm sections. Consecutive slides of each clot were stained with H&E as well as immunostained for CD42b (glycoprotein Ib, rabbit monoclonal anti-CD42b, Abcam ab227669) to identify platelets, von Willebrand factor (monoclonal mouse anti-human vWF, Dako M0616), fibrin/fibrinogen (mouse monoclonal anti-fibrinogen, Abcam ab58207), plasminogen activator inhibitor-1 (rabbit polyclonal anti-PAI-1, Abcam ab66705) and TAFI (rabbit polyclonal antibody Carboxypeptide B2, AP17235PU, Origene). Immunohistochemistry was performed on a Leica Bond Max Autostainer using a RedMap kit (Bond Polymer Refine Red Detection, Leica Biosystems). Representative slides from the patient and control group can be seen in Figs. 1 and 2 [see uploaded Figs. 1 and 2, legend at end].

Stained slides underwent whole slide scanning (Motic Easyscan Pro, Motic Digital Pathology) at 20x magnification. Histological quantification was performed using Orbit Image Analysis Software (www.orbit.bio). The details of the methodology of quantitative analysis using Orbit have been previously described [9, 10].

### Statistical analysis

Data were analyzed using the IBM SPSS-28 software. Data were normally distributed as tested by Kolmogorov-Smirnov test and Q-Q plots so, independent t-test was used to compare patient and control groups. The level of statistical significance for analysis was set at *p* < 0.05 (two-sided). Results were reported as mean ± SD.

## Results

### Clinical characteristics

Relevant clinical data are presented in Tables 1 and 2. The mean age of the high estrogen state patients at the time of stroke was lower than the control group 46.8 ± 15.6 years compared to 52.6 ± 12 years, respectively. The NIHSS was higher for the high estrogen group than the controls 14.5 ± 9.0 compared to 5.0 ± 2.8, respectively. The mean time to recanalize was significantly higher in the high estrogen state group compared to the control group 57.8 ± 27.6 min compared to 22.6 ± 11.4 min, respectively (*p* = 0.0351*). The mean number of passes required was also significantly higher in the high estrogen group compared to controls 4.6 (2.3) passes compared to 1.2 (0.5), respectively, (*p* = 0.0261*). TICI 2b/3 was achieved in nine out of the ten considered cases. Tissue plasminogen activator (tPA) was administered in 2 cases for each group.

**Table 1:** Baseline characteristics of high estrogen state and control groups

	Case	Relevant medical history/co-morbidities	Vascular risk factors	Relevant family history	Medications at the time of stroke
**High estrogen state group**	1	COPD, migraines, s/p hysterectomy, s/p appendectomy, s/p small bowel obstruction	None	N/A	Nitrates, aspirin, Diltiazem, Premarin
	2	Bicuspid aortic valve with severe aortic regurgitation, 5 years s/p prosthetic valve, obese	Hypertension	N/A	Warfarin, oral contraception
	3	None	None	Non-fatal stroke (father), fatal pulmonary embolism (sister)	Oral contraception
	4	Admitted for ruptured MCA pseudoaneurysm	None	N/A	Oral contraception
	5	One-week post-partum, tetralogy of Fallot repaired in infancy with closure of VSD, s/p pulmonary valve replacement	None	N/A	None
**Control group**	1	Prior left cerebellar infarction, s/p medulloblastoma resection and radiotherapy, generalized brain atrophy, history of seizures, thyroiditis	Hypertension	Thyroid disease (mother, maternal grandmother)	None
	2	Tested positive for COVID-19 at the time of admission (with possible ivermectin use), ADHD, Unintentional weight loss (70 lbs), former smoker	Hypertension, hyperlipidemia, DM (hyperglycemia at admission)	N/A	None
	3	History of CAD, anxiety, recent community-acquired pneumonia	Hypertension, hyperlipidemia	N/A	Aspirin, atorvastatin, hydrochlorothiazide
	4	None, former smokeless tobacco user	Hypertension, DM, hyperlipidemia	Stroke (uncle), Hypertension, DM (father), breast cancer, pancreatic cancer, lymphoma, Crohn’s disease (mother), heart disease (grandmother, uncle)	atorvastatin, metformin, lisinopril
	5	Recent AL amyloidosis diagnosis with related cardiomyopathy, PFO, history of BPH, depression	None	N/A	Levothyroxine, finasteride

s/p = status post, COPD = chronic obstructive pulmonary disease, DM = Diabetes mellitus, PFO = patent foramen ovale, VSD = ventricular septal defect, ADHD = attention deficit hyperactivity disorder, CAD = coronary artery disease, BPH = benign prostate hyperplasia, AL = light chain amyloidosis, N/A = not applicable

**Table 2:** Intervention characteristics of high estrogen state and control groups

	Case	Vessel occluded	NIHSS	tPA	Time to recanalize	Number of passes	TICI
**High estrogen state group**	1	Right ICA	18	Yes	31	3	3
	2	Left MCA (M1)	19	No	90	5	2b
	3	Left MCA (M1)	20	No	71	8	3
	4	Left MCA (M2)*	n/a	n/a	n/a	2	n/a
	5	Right MCA (M2)	1	Yes	39	5	3
**Control group**	1	Mid-basilar artery	n/a	No	12	1	3
	2	Right MCA (M2)	n/a	No	12	1	2b
	3	Basilar artery extending to left P1	3	Yes	22	1	3
	4	Right vertebral and proximal basilar artery	7	No	39	multiple	2a
	5	Right inferior M2 occlusion	n/a	No	28	2	3

*Intraprocedural thrombus.

ICA = Internal Carotid Artery; MCA = Middle Cerebral Artery; NIHSS = National Institutes of Health Stroke Scale; tPA = tissue Plasminogen Activator; TICI = Thrombolysis in Cerebral Infarction; N/A = not available.

### Histological composition of thrombi

Immunostaining for TAFI using Carboxypeptide B2 stain showed the expression of TAFI to be significantly higher in the hyperestrogenic clots than in controls with mean expression 25.6 ± 11.9% compared to 9.3 ± 9.0%, respectively (*p* = 0.043*). CD42b expression using anti-CD42b immunostain was found to be significantly lower in the hyperestrogenic clots than in the control group with mean expression 41.7 ± 10.6% compared to 61.7 ± 12.9%, respectively (*p* = 0.029*). We did not find any significant difference in the expression of vWF, Fibrinogen or PAI-1. Quantification of anti-vWF stain showed a mean expression of 27.2 ± 17.0% in hyperestrogenic clots and 35.0 ± 20.7% in controls (*p* = 0.536). The mean expression of fibrinogen using anti-fibrinogen stain was 43.8 ± 8.9% in hyperestrogenic clots and 55.3 ± 15.6% in controls (*p* = 0.200). The quantification of anti-PAI-1 stain showed a mean expression of 41.1 ± 20.3% in hyperestrogenic clots and 61.8 ± 10.4% in controls (0.172). Quantification of each component is given in Table 3.

**Table 3:** Quantification of histological composition of retrieved thrombi

	High estrogen state group	Control group	*P* value
TAFI (%) Mean ± SD	25.55 ± 11.89	9.31 ± 9.00	0.043*
CD42b (%) Mean ± SD	41.73 ± 10.58	61.77 ± 12.93	0.029*
vWF (%) Mean ± SD	27.20 ± 17.03	34.97 ± 20.70	0.536
Fibrinogen (%) Mean ± SD	43.79 ± 8.87	55.25 ± 15.56	0.200
PAI-1 (%) Mean ± SD	41.13 ± 20.26	61.80 ± 10.35	0.172

TAFI = Thrombin activatable fibrinolysis inhibitor; vWF = von Willebrand factor; PAI-1 = Plasminogen activator inhibitor-1

## Discussion

In the present case-control study we performed a histological and immunohistochemical analysis of clots retrieved from AIS patients in high estrogen states. We found that TAFI expression was significantly higher in hyperestrogenic thrombi compared to controls, whereas platelet (CD42b) expression was significantly higher in the control group. Upon analysis of the other clot components, we found a no significant difference in the expression of of PAI-1, vWF and fibrinogen in the hyperestrogenic thrombi compared to controls. These findings are important because our study demonstrates a possible interplay of TAFI and high estrogen states in the occurrence of stroke in a specific subset of patients, both factors being known to increase the risk of AIS.

TAFI has been considered an important factor in the delicate balance between coagulation and fibrinolysis [7], and a number of studies have described TAFI as a risk factor for ischemic stroke, with increased levels being found in AIS patients [6–8]. Notably, TAFI prevents tPA-induced activation of plasminogen [7, 8] thus retarding clot lysis times, and previous studies reported that tPA concentration needs to be increased 7-8-fold to achieve the same clot lysis times when TAFI levels are maximal [11–13]. Moreover, previous studies have described that increased TAFI activity by OCP use may induce changes of the fibrinolytic pathway [4, 16]. The increased TAFI activation compensates for the profibrinolytic effect of OCPs by downregulating fibrinolysis and therefore, protecting the clot from lysis [5, 14]. Further, it has been described that platelet-derived TAFI represents a very small percentage (0.1%) of total TAFI found in plasma and thus platelet-rich clots may be conferred with resistance to fibrinolysis via local TAFI secretion [15]. However, the anti-fibrinolytic activity of TAFI has been recently attributed to plasma-derived and not platelet-derived TAFI [16].

Interestingly, we also found the patient suffering from an AIS one-week post-partum had the lowest levels of thrombus TAFI. Mousa et al. found TAFI levels and, thus, clot lysis time increase during pregnancy, peaking towards the end of term. They described TAFI levels returning to normal rapidly within 24 h of delivery, whereas other coagulation factors take longer, up to weeks, to normalize [17].

The prothrombogenicity of estrogens through changes in hepatic coagulation factor production is well-documented [1, 3]. However, the relationship between estrogens and platelet production and function (activation and aggregation) remains unclear and the molecular mechanisms are thus far poorly investigated. Reviews considering human and mouse studies are unable to completely characterize the relationship or effect of hormonal therapy on platelet behaviour and describe it as “highly variable” and difficult to elucidate due to contradictory results in the studies reviewed [4, 18].

Additionally, the time to recanalize and number of passes required were also significantly higher in the high estrogen state group in comparison to the control group. This finding suggests that the clots retrieved from the high estrogen state group were possibly stickier and harder retrieve, thus requiring more time and/or a higher number of passes for successful revascularization. Tougher clots may be attributed to TAFI’s ability to make clots more resistant to breakdown resulting in longer procedural times and more complicated thrombectomies, however this hypothesis requires further exploration.

Our study has limitations. Firstly, the small number of patients and controls due to the highly niche parameters being assessed. Second, the inhomogeneous use of exogenous estrogen under different conditions may result in different composition of thrombi. Third, plasma TAFI levels were not measured in these patients; only thrombus TAFI was quantified. An additional limitation is the lack of some clinical data. Lastly, this study was a single-center study and thus, our findings may not be generalizable.

Further studies exploring cerebral thrombi in high estrogen states would be useful in understanding the structure of these specific clots and the difference from other groups, if any. Larger cohort studies are required to confirm our hypothesis of histological difference in clots formed in high estrogen states and the role of TAFI and its relationship with OCPs and AIS.

## Conclusions

In conclusion, our study showed a significantly higher expression of TAFI in stroke thromboemboli among patients with high estrogen states compared to controls. Larger cohort studies are required to confirm our results and further investigate the structure of these specific clots as well the role of TAFI and estrogen in AIS pathogenesis.

## Data Availability

Data are available from the corresponding author upon reasonable request.
